# Left Atrial Strain in Cardiomyopathies and Congenital Heart Disease: A Call for Its Integration into Clinical Practice

**DOI:** 10.3390/jcm12155084

**Published:** 2023-08-02

**Authors:** Giulia Pelaia, Daniela Concolino, Jolanda Sabatino

**Affiliations:** 1 Paediatric Unit, Department of Science of Health, Magna Graecia University, 88100 Catanzaro, Italy; giulia.pelaia@gmail.com (G.P.); dconcolino@unicz.it (D.C.); 2 Paediatric Cardiology and Congenital Heart Disease Unit, Department of Experimental and Clinical Medicine, Magna Graecia University, 88100 Catanzaro, Italy; 3 Paediatric Research Institute (IRP), Città Della Speranza, 35127 Padua, Italy

Despite the long-standing neglect, there is now a mounting interest in the left atrium (LA) physiology.

Nowadays, it is widely acknowledged that LA coordinates the left ventricular (LV) filling and the cardiovascular systo-diastolic functions through its roles as a reservoir, conduit, and booster pump. During ventricular systole, it serves as a reservoir for pulmonary venous return, while in early ventricular diastole, it acts as a conduit for pulmonary venous return. During late ventricular diastole, it also functions as a booster pump, enhancing ventricular filling.

The interdependent nature of the LA functions serves as a compensatory mechanism, aiding in the facilitation of LV filling in individuals with myocardial disease. Moreover, the atrial reservoir phase plays a crucial role in LV filling as it accumulates energy during the ventricular systole, which is subsequently discharged upon the opening of the mitral valve.

Notwithstanding the availability of a wide range of echocardiographic and Doppler techniques for measuring LA function, the preferred method is typically the speckle-tracking echocardiography (STE) strain analysis, which captures both the global and regional deformation of the myocardium by tracking the natural acoustic markers (speckles) produced through the interaction between ultrasound waves and the myocardial tissue on a frame-by-frame basis.

Understanding the LA deformation plays a critical role in congenital heart disease and cardiomyopathies (especially in pediatric age) where there is an unmet need to anticipate left myocardial failure on a subclinical basis.

The employment of left atrial (LA) strain is still poorly understood in children, although it is known to be helpful for the assessment of diastolic function in adults. For this purpose, Sabatino et al. [1] investigated the accuracy of LA strain for the diagnosis of diastolic dysfunction in children with cardiomyopathies, inquiring how the left atrium reservoir, conduit, and booster functions can be related to left ventricle filling also in a pediatric population. The study consists of the evaluation of 136 children with dilated (CMD), hypertrophic (HCM), and restrictive (RCM) cardiomyopathies using standard transthoracic echocardiography and two-dimensional (2-D) speckle-tracking analyses. The study results led to the understanding that LA strain and strain values are more accurate to recognize diastolic dysfunction early despite the other conventional parameters analyzed in other studies, such as E/E’, mitral E wave deceleration time (DT), and left atrial volume indexed to body surface area (LAVi). Moreover, unlike the LA strain, these conventional measurements are unable to highlight differences between distinct subgroups of cardiomyopathies. Another important novelty underlined by this paper consists of affirming, unprecedently in children, the correlation between LA strain and peak tricuspid regurgitation gradient, which is higher in patients with HCM and RCM. Furthermore, additional new findings confirm an inverse and strong correlation between LA strain values and LV end-diastolic pressures also in pediatric age.

New insights derived from LA strain assessment in congenital heart diseases have been set up by Nemes et al. [2] in their study on adult patients affected by dextro-transposition of the great arteries (dTGA), who underwent Mustard or Senning surgery procedures. In this case, the method used to perform the strain evaluation is the three-dimensional (3D) speckle-tracking echocardiography (3DSTE). This work’s aim embraces the need for additional echocardiographic parameters to correctly follow up patients with dTGA undergoing surgery in childhood. Even if the study population is not very large, all cases presented relevant abnormalities in LA strain and, then, in the global LA function compared with healthy subjects. This kind of data about LA anomalies have been obtained in different types of pathologies, but dTGA adult-operated patients have never been studied in depth from this point of view. More precisely, atrial function and contractility were previously tested in dTGA after switch repair, but only through MRI and never by an echocardiographic assessment. According to Nemes et al., the majority of Mustard-operated patients exhibited lower peak left atrial strains in comparison to Senning-operated individuals, which indicates that the Senning procedure appears to have a more favorable long-term effect on left atrial volumetric and functional characteristics compared to the Mustard procedure.

An interesting use of the LA strain was implemented by Esposito et al. [3], which analyzed the feasible correlation between LA dysfunction and white matter lesions (WMLs) from the central nervous system in patients with Anderson–Fabry disease (AFD). The pathogenesis of AFD involves the progressive deposition of sphingolipids in multiple organs, including the heart and cardiomyocytes, causing complications such as arrhythmias and hypertrophic cardiomyopathy. Among these, atrial fibrillation (AF) should not be underestimated, as it can produce neurological conditions from rarely symptomatic WMLs to more severe ischemic strokes. Therefore, this study sought to explore the possible link joining LA strain and WMLs, since the current literature is lacking in this regard. Peak atrial longitudinal strain (PALS), a reliable index of atrial strain, was evaluated by speckle tracking echocardiography, while WMLs were identified through brain MRI, combining FLAIR and DWI sequences, and estimating the so-called Fazekas’ Score (FS). Higher values of this parameter are related to more severe hyperintense lesions localized on periventricular and deep white matter. Previously, other works have shown that a reduction in PALS can be observed in the general population with recurrent AF and also in AFD patients, but it has never been considered as a tool to link possible cardiological and neurological complications of AFD in patients not yet undergoing enzyme replacement treatment. The results highlight an inverse proportionality relationship between PALS and FS; finding a marked reduction in PALS is statistically associated with a strongly increased FS. Then, PALS, in addition to being an early marker of cardiovascular involvement in AFD, also represents the only echocardiographic parameter that can be directly related to the onset of neurological complications, helping to classify patients according to FS. Thanks to this study, we now know that PALS anomalies will suggest the execution of brain MRI for rapid detection of neurological involvement, and, above all, it will lead to an early start of enzyme replacement therapy to block the glycosphingolipids storage.

Currently, atrial speckle tracking echocardiography strain has been poorly studied in the pediatric population undergoing cardiac surgery. For this purpose, Cantinotti et al. [4] presented interesting and innovative results in evaluating right and left atrial strain as an index of cardiac dysfunction in the post-operative phase in children with congenital heart disease (CHD). All the patients enrolled received biventricular cardiac surgery and subsequently echocardiograms at three distinct times post-procedure: after 24–36 h, 3–5 days, and 6–9 days (respectively, Time 1, 2, and 3). Specifically, this study’s goal is to define the utility and reliability of the atrial strain as a tool for progressive post-operative evaluation in three categories of patients affected by CHD (neonates, infants, and older children), comparing them with same-aged healthy subjects. The analysis shows that all the pre-operative data acquired from CHD patients were inferior to controls, but the lowest values were found at the first post-surgery echocardiographic determination. The same trend was observed at Time 3, excluding the data that referred to the LA conduit strain function, much closer to control results than the other comparisons. Generally, a progressive improvement of all the atrial parameters evaluated should be noted. In particular, the first variable that showed a significant increase between one detection and the next is the reservoir bilateral atrial strain function. A further important content deduced from this work is represented by the close correlation between atrial and ventricular strain, and consequently with left ventricular ejection fraction. This study used a new technique that made it possible to estimate atrial strain according to two methods, P-gating and R-gating, which are not interchangeable, but both reliable. Moreover, since using this software is easily executable, it is advisable to extend its use as a routine examination before surgical intervention in children with CHD.

We have observed that studies on the echocardiographic evaluation of strain in pediatric age are progressively increasing; however, this is not the only method that can be used to evaluate atrial deformation. Stojanovska et al. [5] investigated how left atrial function in patients with restrictive cardiomyopathy (RCM) relates to the probability of experiencing an adverse event responsible for hospitalization or death. To achieve this aim, cardiac magnetic resonance (CMR) was performed on all subjects involved and late gadolinium enhancement (LGE) was employed as a marker to identify myocardial remodeling and also to classify different types of RCM. Substantially, this work has demonstrated how the LA strain, especially the LA reservoir strain, allows the early recognition of which RCMs are most at risk of experiencing adverse events, without the need to resort to other indices such as LGE.

When compared to the LV, strain imaging of the left atrium (LA) is considered more challenging and time-consuming among most cardiologists and echocardiographers. This is primarily due to several factors, including the far-field location and the thin walls of the atrium, the presence of the appendage and the pulmonary veins, and the requirement for obtaining non-foreshortened views of the atrium.

Nevertheless, there are compelling reasons to utilize LA strain in the clinical setting. Firstly, LA strain combines multiple parameters into a single reliable and reproducible measurement. Secondly, LA strain has demonstrated significant diagnostic value and an ability to predict cardiovascular events, particularly in patients with cardiomyopathies and congenital heart disease, although its applicability is not limited to these conditions.

Considering the extensive research conducted on this parameter, it is now imperative to integrate it into diagnostic stratification strategies and consensus documents.
